# Factors predicting lower limb alignment after Oxford medial unicompartmental knee arthroplasty

**DOI:** 10.1038/s41598-024-56285-x

**Published:** 2024-03-07

**Authors:** Han-Ting Shih, Kun-Hui Chen, Cheng-Hung Lee, Kao-Chang Tu, Shun-Ping Wang

**Affiliations:** 1 Department of Orthopaedics, Taichung Veterans General Hospital, 1650 Taiwan Boulevard Sect. 4, Taichung, 40705 Taiwan; 2 Department of Post‐Baccalaureate Medicine, College of Medicine, National Chung Hsing University, Taichung, Taiwan; 3 Department of Computer Science and Information Engineering, Providence University, Taichung, Taiwan; 4 Department of Food Science and Technology, HungKuang University, Taichung, Taiwan

**Keywords:** Musculoskeletal system, Reconstruction

## Abstract

This study aimed to identify the factors affecting hip−knee−ankle (HKA) angle following Oxford medial unicompartmental knee arthroplasty (MUKA). A retrospective analysis of 200 patients who underwent Oxford MUKA from June 2018 to October 2020 was conducted. Univariate and multivariate analyses were performed to investigate the impact of surgical and radiographic characteristics on the postoperative HKA angle. The mean HKA angle was 9.5 ± 4.3° before surgery and 3.6 ± 3.7° after surgery (*p* < 0.001). The postoperative HKA angle significantly correlated with the preoperative HKA angle, bearing size, tibial component alignment angle, and BMI (*r* = 0.71, *p* < 0.001; *r* =  − 0.24, *p* = 0.001; *r* = 0.21, *p* = 0.004; *r* =  − 0.18, *p* = 0.011). Multiple linear regression analysis revealed that the preoperative HKA angle (*β* = 0.68, *p* < 0.001), bearing size (*β* =  − 0.31, *p* < 0.001), tibial component alignment angle (*β* = 0.14, *p* = 0.003), and BMI (*β* =  − 0.09, *p* = 0.047) significantly affected the postoperative HKA angle. In conclusion, larger preoperative varus deformity, smaller bearing size, greater varus alignment of the tibial component, and lower BMI lead to greater postoperative varus alignment of the lower limb in Oxford MUKA. With this concept, surgeons can more accurately predict postoperative lower limb alignment and avoid malalignment in Oxford MUKA.

## Introduction

With the aging of the population, an increasing number of individuals are afflicted by degenerative joint disease in the knee. Consequently, a growing cohort is undergoing knee joint replacement surgery^1^. In pursuit of smaller incisions, reduced soft tissue damage, and expedited postoperative recovery, there is a rising demand for minimally invasive procedures, encompassing minimally invasive total knee arthroplasty (TKA) and unicompartmental knee arthroplasty (UKA). Oxford medial unicompartmental knee arthroplasty (MUKA), a mobile-bearing partial knee replacement, is commonly employed for the treatment of osteoarthritis and osteonecrosis in the medial compartment of the knee^2,3^. With recent enhancements in design, the Oxford MUKA can provide considerably satisfactory clinical outcomes, long-term implant survival, and patient satisfaction, and is therefore being more widely adopted^3–6^; however, a noteworthy concern revolves around its elevated revision rate compared with TKA and fixed-bearing UKA^5,7^. Therefore, further research is needed to explore the reasons behind this.

Oxford MUKA is referred to as a resurfacing surgery primarily aimed at pain relief. Despite ongoing debate in the current literature, correcting lower limb alignment is not the primary objective of Oxford MUKA, and achieving proper lower limb alignment does not necessarily guarantee superior clinical outcomes^8,9^. Patients typically exhibit varying degrees of lower limb varus alignment preoperatively due to different functional knee phenotypes and varying disease severity^10^. In clinical practice, significant changes in lower limb alignment are frequently observed after Oxford MUKA^11–14^, which can alter lower limb musculoskeletal biomechanics and may potentially influence implant longevity^15–19^. Postoperative varus malalignment (i.e., excessive undercorrection) may accelerate polyethylene wearing and recurrence of lower limb deformity^15–17^, whereas postoperative valgus malalignment (i.e., excessive overcorrection) may accelerate lateral compartment arthritis and lead to a higher revision rate^16,18,19^. Therefore, accurately predicting the postoperative mechanical axis in Oxford MUKA is critical.

Although many studies have identified the factors affecting lower limb alignment after Oxford MUKA, the results remain controversial^11,14,20,21^. Most of the existing studies have focused on the risk factors contributing to the postoperative lower limb malalignment^14,21^. However, due to the presence of various preoperative functional knee phenotypes and disease severity, a consensus on the clear definition of malalignment after Oxford MUKA is still lacking^10^. Furthermore, these studies have not definitively identified the direct influencing factors on postoperative lower limb alignment. The hip−knee−ankle (HKA) angle, widely utilized in clinical practice to represent lower limb alignment, plays a crucial role in achieving proportional load distribution between the medial and lateral compartments of the knee joint^22^. Therefore, the current study investigated the factors directly influencing the HKA angle after Oxford MUKA. On the other hand, few studies have investigated the effects of the size and position of components on postoperative lower limb alignment. This study comprehensively analyzes these factors, with an aim to enable surgeons to consider all the influential factors and facilitate better postoperative lower limb alignment. In addition to carefully evaluating these factors and selecting the proper surgical indications preoperatively, surgeons must also make cautious adjustments intraoperatively to avoid worsening the lower limb malalignment.

The aim of this study was to explore the factors affecting the HKA angle after the Oxford MUKA. It was hypothesized that patients’ demographic characteristics, surgical characteristics, and the size and position of components could impactthe postoperative HKA angle.

## Methods

### Patient enrollment

This study was conducted in compliance with ethical standards and guidelines, and received approval from the Institutional Review Board (IRB) of Taichung Veterans General Hospital (TCVGH), with IRB No. CE22468B. Informed consent for this retrospective study was waived due to the utilization of extracted data from medical records, which was also reviewed and approved by the IRB of TCVGH. Patients who had had osteoarthritis or osteonecrosis at the medial compartment of the knee and underwent Oxford MUKA (Zimmer Biomet, Warsaw, IN, USA) between June 2018 and October 2020 and had received preoperative and postoperative full-length standing anteroposterior radiographs were recruited. The surgeries were performed by four experienced surgeons who have had many years of experience in performing Oxford MUKA surgeries. According to Goodfellow et al. and Svärd et al. ^23,24^, the indications for performing Oxford MUKA include the following: (1) functionally intact anterior and posterior cruciate ligaments; (2) well-preserved lateral compartment, with full-thickness articular cartilage and intact meniscus; (3) varus deformity < 15°, with correctable varus deformity at 20° flexion; and (4) flexion deformity < 15°. Patients with a history of ipsilateral lower limb fracture or surgery, signs of extraarticular deformity, congenital developmental abnormalities, autoimmune disease, incomplete medical or imaging records, or severe deformity that was difficult to measure were excluded. These extraarticular deformities can simultaneously impact both the sagittal and axial planes, deviating from the normal leg's load transmission. This study exclusively focuses on coronal plane analysis, excluding such scenarios. The progression of congenital developmental abnormalities and autoimmune diseases is continuous. Challenges in research control arise from the varying disease activity and treatment history, leading to their exclusion. All data on patient demographics and surgical characteristics were collected from electronic medical records. A total of 301 patients underwent Oxford MUKA during the enrollment period, among whom 212 had both preoperative and postoperative full-length standing anteroposterior radiographs. Twelve patients were excluded due to the aforementioned exclusion criteria, including four individuals with a history of ipsilateral lower limb fracture or surgery, two with signs of extraarticular deformity, one with congenital developmental abnormalities, one with autoimmune disease, two with incomplete medical or imaging records, and two with severe deformity that was difficult to measure. Finally, 200 patients (143 women [71.5%] and 57 men [28.5%]) were recruited, with a mean age of 67.6 ± 7.7 years and a mean body mass index (BMI) of 28.0 ± 3.5 kg/m^2^. The mean follow-up duration of their full-length standing anteroposterior radiographs was 36.0 ± 6.1 days. Their demographic data are summarized in Table 1.

**Table 1:** Patient characteristics.

	(N = 200)	
Age (years)	67.6	± 7.7
BMI (kg/m^2^)	28.0	± 3.5
Gender		
Female	143	(71.5%)
Male	57	(28.5%)
Diagnosis		
Osteoarthritis	191	(95.5%)
Osteonecrosis	9	(4.5%)
Side		
Right	102	(51.0%)
Left	98	(49.0%)
Surgical approach		
Subvastus	130	(65.0%)
Midvastus	62	(31.0%)
Medial parapatellar	8	(4.0%)
Size of femoral component		
Small	91	(45.5%)
Extra small	56	(28.0%)
Medium	46	(23.0%)
Large	7	(3.5%)
Size of tibial component		
B	70	(35.0%)
A	47	(23.5%)
C	44	(22.0%)
D	25	(12.5%)
AA	14	(7.0%)
Size of meniscal bearing		
3 mm	116	(58.0%)
4 mm	46	(23.0%)
5 mm	29	(14.5%)
6 mm	9	(4.5%)

Categorical data were expressed as number (percentage).

Continuous data are presented as mean ± SD.

### Surgical procedure

Patients were operated on under general or neuraxial anesthesia. The affected lower limb was placed on thigh support to set the hip joint at 30°. The flexion of the knee joint was confirmed to be at least 110°. The entire surgical procedure was completed with the use of a tourniquet. The surgical approaches applied (i.e., the subvastus, midvastus, and medial parapatellar approaches) depended on the surgeon’s preference. Upon opening the knee joint cavity, thorough inspection was conducted to assess the entire medial compartment, the integrity of the cruciate ligaments, and articular cartilage of the lateral femoral condyle. During the entire procedure, the surgeon avoided releasing the medial collateral ligament and cleared all visible osteophytes. The resection of the medial tibial plateau was performed with the assistance of an extramedullary alignment guide so that it was perpendicular to the mechanical axis of the tibia on the coronal plane. The posterior resection of the femur was performed with the assistance of an intramedullary rod and link. The distal femoral was milled using a spigot system until the equality of the flexion and extension gaps was confirmed. The femoral and tibial components were fixed to the bone with cement. Gradually thickened trial meniscal bearings of 1 mm increments were utilized, employing the "lift-off technique" to evaluate tension^25^. A single finger applied force to the trial meniscal bearing's handle, lifting it off the tibial component. The objective was to achieve a uniform 3 mm lift-off in both 90 degrees and 20 degrees of flexion positions with equal effort. Appropriate polyethylene inserts were selected and confirmed to have no excessive tension. Finally, the full range of motion was tested to verify that no bearing impingement, rotation, or dislocation occurred. All patients received the same postoperative care and rehabilitation protocol.

### Radiographic measurements

**Figure 1:**
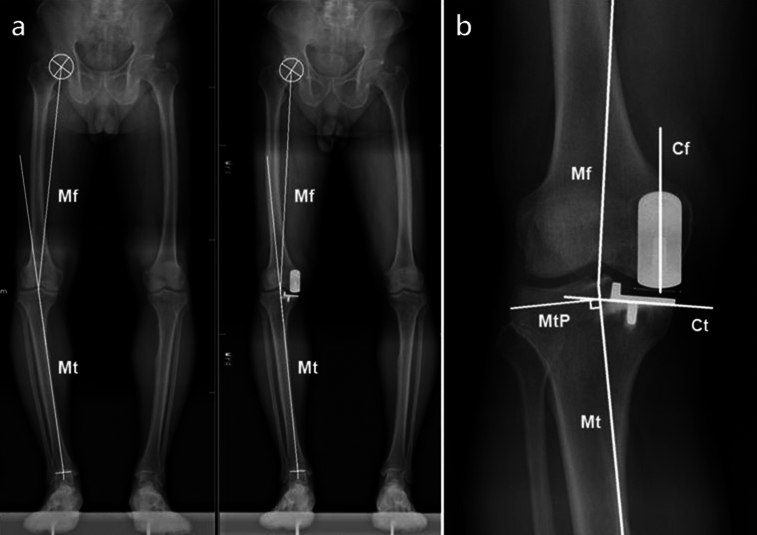
Measurement of preoperative and postoperative hip–knee–ankle angle (**a**) and component alignment angle (**b**) on full-length standing anteroposterior radiographs. The hip–knee–ankle angle was defined as the angle between the Line Mf and the Line Mt. The femoral component alignment angle was defined as the angle between the Line Mf and the Line Cf. The tibial component alignment angle was defined as the angle between the Line MtP and the Line Ct. The varus and valgus alignments of the HKA angle, femoral component, and tibial component were defined as positive and negative, respectively.Line Mf, the mechanical axis of the femur; Line Mt, the mechanical axis of the tibia; Line MtP, the perpendicular line of the mechanical axis of the tibia; Line Cf, the longitudinal axis of the femoral component; Line Ct, the undersurface of the tibial component.

The full-length standing anteroposterior radiographs of the lower limbs that were closest to the days before and after the surgery were selected for measurement. The preoperative HKA angle, postoperative HKA angle, and femoral and tibial component alignment angle were measured. The measurement method is illustrated in Fig. 1**.**

The HKA angle was the angle between the mechanical axis of the femur and the mechanical axis of the tibia. The mechanical axis of the femur was defined as the line connecting the hip center and the knee center. The mechanical axis of the tibia was defined as the line connecting the knee center and ankle center^26–28^. The femoral component alignment angle was defined as the angle between the longitudinal axis of the femoral component and the mechanical axis of the femur, and the tibial component alignment angle was defined as the angle between the undersurface of the tibial component and the perpendicular line of the mechanical axis of the tibia^29–31^. The varus and valgus alignments of the HKA angle, femoral component, and tibial component were defined as positive and negative, respectively.

### Statistic analysis

All statistical analyses were performed using SPSS version 26 (IBM, Armonk, NY, USA). Means and standard deviations were used to describe continuous variables, and frequencies with percentages were used to describe categorical variables. A paired sample *t* test was conducted to compare the preoperative and postoperative HKA angles. The correlation of the postoperative HKA angle with the continuous variables (e.g., age, BMI, preoperative HKA angle, femoral component alignment angle, tibial component alignment angle, and bearing size) was analyzed using Pearson’s correlation coefficients. Between-group differences in the postoperative HKA angle (divided according to sex, diagnosis, the affected side, surgical approach, and the sizes of the femoral and tibial components) were analyzed using an independent samples *t* test or one-way analysis of variance. Finally, simple and multiple linear regression analyses were adopted to examine the factors that possibly affected the postoperative HKA angle (e.g., age, sex, BMI, diagnosis, the affected side, surgical approach, the sizes and alignments of the components, and the preoperative HKA angle). Using G*Power 3 (Heinrich Heine Universität Düsseldorf, Germany), a power analysis was performed to estimate the minimum sample size (N = 150) required for a multiple linear regression analysis (input parameters: effect size = 0.15, α = 0.05, power = 0.80, number of predictors = 18)^32^.

## Results

The demographic data and surgical characteristics are presented in Table 1, while the radiographic results are displayed in Table 2. The mean preoperative HKA angle was 9.5 ± 4.3°, significantly decreasing to 3.6 ± 3.7° after Oxford MUKA (p < 0.001), with a mean correction of 5.9 ± 3.1°.

**Table 2:** Radiologic Results.

	Mean	± SD	*p* value^+^
HKA angle			
Preoperative (°)	9.5	± 4.3	
Postoperative (°)	3.6	± 3.7	
Correction (°)	-5.9	± 3.1	< 0.001**
Component alignment angle			
Femoral (°)	0.8	± 4.2	
Tibial (°)	4.8	± 3.3	

*HKA* hip−knee−ankle. The varus and valgus alignments of the HKA angle, femoral component, and tibial component were defined as positive and negative, respectively.

^+^ Paired sample *t* test of preoperative and postoperative HKA angle. ** *p* < 0.01.

The postoperative HKA angle had a strong positive correlation with the preoperative HKA angle (*r* = 0.71, *p* < 0.001), weak positive correlations with the femoral component alignment angle (*r* = 0.15, *p* = 0.031) and tibial component alignment angle (*r* = 0.21, *p* = 0.004), weak negative correlations with BMI (*r* =  − 0.18, *p* = 0.011) and bearing size (*r* =  − 0.24, *p* = 0.001), and no correlation with age (Table 3).

**Table 3:** Correlations between postoperative HKA angle and patient characteristics.

	Postoperative HKA angle	
	*r*	*p* value^+^
Age	− 0.07	0.299
BMI	− 0.18	0.011*
Size of meniscal bearing	− 0.24	0.001**
Preoperative HKA angle	0.71	<0.001**
Femoral component alignment angle	0.15	0.031*
Tibial component alignment angle	0.21	0.004**

*HKA* hip−knee−ankle. The varus and valgus alignments of the HKA angle, femoral component, and tibial component were defined as positive and negative, respectively.

^+^ Pearson’s correlation coefficients. * *p* < 0.05, ** *p* < 0.01.

Grouping was conducted according to sex, diagnosis, the affected side, surgical approach, and the sizes of the femoral and tibial components. No significant between-group differences were observed in the postoperative HKA angles.

The simple linear regression analysis revealed that 6 of 12 variables likely affected the postoperative HKA angle (*p* < 0.05): BMI, femoral component size, bearing size, preoperative HKA angle, femoral component alignment angle, and tibial component alignment angle. Among them, multiple linear regression analysis revealed that BMI (*β* =  − 0.09, *p* = 0.047), bearing size (*β* =  − 0.31, *p* < 0.001), preoperative HKA angle (*β* = 0.68, *p* < 0.001), and tibial component alignment angle (*β* = 0.14, *p* = 0.003) significantly affected the postoperative HKA angle (Table 4).

**Table 4:** Univariate and multivariate analysis of postoperative HKA angle.

	Simple linear regression analysis			Multiple linear regression analysis		
	B	Beta (β)	*p* value	B	Beta (β)	*p* value
Age	− 0.04	− 0.07	0.299			
BMI	− 0.19	− 0.18	0.011*	− 0.09	− 0.09	0.047*
Gender						
Female	ref					
Male	0.83	0.10	0.149			
Diagnosis						
Osteoarthritis	ref					
Osteonecrosis	− 0.69	− 0.04	0.582			
Side						
Right	ref					
Left	0.31	0.04	0.558			
Surgical approach						
Subvastus	ref					
Midvastus	0.05	0.01	0.929			
Medial parapatellar	− 0.80	− 0.04	0.553			
Size of femoral component						
Small	ref			ref		
Extra small	1.50	0.18	0.016*	0.75	0.09	0.061
Medium	0.27	0.03	0.685	− 0.24	− 0.03	0.570
Large	1.80	0.09	0.209	0.46	0.02	0.618
Size of tibial component						
B	ref					
A	− 1.16	− 0.13	0.096			
C	− 0.74	− 0.08	0.298			
D	0.72	0.06	0.402			
AA	− 0.37	− 0.03	0.732			
Size of meniscal bearing	− 1.00	− 0.24	0.001**	− 1.28	− 0.31	< 0.001**
Preoperative HKA angle	0.61	0.71	< 0.001**	0.58	0.68	< 0.001**
Femoral component alignment angle	0.14	0.15	0.031*	0.04	0.05	0.291
Tibial component alignment angle	0.23	0.21	0.004**	0.15	0.14	0.003**
	Adjusted R^2^			60.99%		

*HKA* hip−knee−ankle. The varus and valgus alignments of the HKA angle, femoral component, and tibial component were defined as positive and negative, respectively.

* *p* < 0.05, ** *p* < 0.01.

## Discussion

The study findings indicated that BMI, bearing size, preoperative HKA angle, and tibial component alignment angle predicted the postoperative HKA angle, with preoperative HKA angle having the strongest predictive effect, followed by bearing size, tibial component alignment angle, and BMI. Greater varus alignment of the preoperative lower limb and tibial component, smaller meniscal bearing, and thinner patient render the postoperative lower limb alignment even more varus. Surgeons meticulously consider these influencing factors both preoperatively and intraoperatively, contributing to improved accuracy in predicting postoperative alignment and preventing lower limb malalignment.

The alignment after Oxford MUKA considerably affects its prognosis. Although the target value of the HKA angle after Oxford MUKA remains inconclusive, an increasing number of studies recommend slight undercorrection of the lower limb deformity^18,33–36^. A finite element analysis showed that compared with a neutral or 3°-valgus lower limb alignment, a 3°-varus lower limb alignment after surgery had lower contact stresses and load percentages in the lateral compartments^33^. Clinically, patients with slight undercorrection are less prone to lateral compartment degeneration^18,34^ and have higher functional scores^35,36^. Furthermore, excessive varus undercorrection or excessive valgus overcorrection can result in a poor prognosis^15–17,19^. Excessive varus undercorrection can accelerate polyethylene wearing and recurrence of the deformity^15–17^, whereas excessive valgus overcorrection may lead to degeneration in the lateral compartments^16,18,19^. Both cause early failure and revision surgery. Therefore, the issue of accurately predicting the postoperative lower limb alignment in Oxford MUKA prompted the initiation of this research.

In this study, preoperative HKA angle and bearing size were the two major factors affecting the postoperative HKA angle. Their standardized coefficients (*β*) were 0.68 and − 0.31, respectively. This finding is consistent with the conclusions of many studies^11,20,37,38^. Mullaji et al. analyzed the factors affecting the postoperative HKA angle, including age, sex, BMI, preoperative HKA angle, insert thickness, and the surgeon’s experience, and discovered that only the preoperative HKA angle affected the postoperative HKA angle^11^. Mullaji et al. and Tashiro et al. have revealed that the preoperative HKA angle had a significantly moderate-to-strong positive correlation with the postoperative HKA angle^11,37^, which implies that a severe varus deformity preoperatively predisposed the lower limb to become excessively varus postoperatively. This may be because severe preoperative varus deformity may be accompanied by medial collateral ligament contracture, and the medial collateral ligament is generally not released during the Oxford MUKA procedure. Therefore, the medial compartment space remains narrow. On the other hand, another significant factor influencing the postoperative HKA angle is the bearing size. It is intricately linked to the extent of HKA angle correction, impacting the postoperative functional outcomes of Oxford MUKA. The literature suggests that lesser HKA angle correction may lead to postoperative pain, stiffness, and even revision surgery^15^. Hopgood et al. and Kim et al. have implied that the amount of HKA angle correction was significantly different under different bearing sizes^20,38^. A larger bearing size can contribute to a larger amount of HKA angle correction, and the lower limb alignment becomes more valgus after the surgery. The selection of the bearing size is based on the surgeon’s assessment of the tension of the ligament during surgery. However, these assessments can be subjective. The surgeon may sometimes select a thicker-than-ideal meniscal bearing to avoid instability or even bearing dislocation, which is one of the most common complications of revision surgery^39,40^. However, the results of this study are inconsistent with the findings of several other studies. Ahn et al. and Zhang et al. reported that both the preoperative HKA angle and the bearing size were not risk factors for postoperative lower limb malalignment^14,41^. The conflicting and inconsistent results in the literature were part of the motivation for the present investigation.

The current study found that the tibial component alignment angle and BMI also affected the postoperative HKA angle, which is inconsistent with previous studies^14,20,21,41^. Ahn et al. and Zhang et al. revealed that the tibial component alignment angle was not a risk factor for postoperative lower limb malalignment^14,41^. Kim et al. also noted that the amount of HKA correction would not change with the tibial resection angle^20^. Furthermore, three studies indicated that BMI was not a risk factor for postoperative lower limb malalignment^14,21,41^. These discrepancies may be because the aforementioned studies applied the endpoints to determine postoperative lower limb malalignment, rather than directly inspecting the predictive factors of postoperative HKA angle. Currently, the definition of lower limb optimal alignment and malalignment after Oxford MUKA is unclear and controversial, leading to heterogeneity among study results. This study directly analyzed the predicting factors for the postoperative HKA angle, which can help surgeons predict postoperative lower limb alignment in a more intuitive manner. Moreover, although the tibial component alignment angle and BMI had a significant effect on the postoperative HKA angle, the effect was mild. Their standardized coefficients (*β*) were 0.14 and − 0.09, respectively. Compared with the literature, the present study had a larger sample size. An a priori power analysis was conducted in advance to ensure a sufficient sample size and adequate power for the analysis.

**Figure 2:**
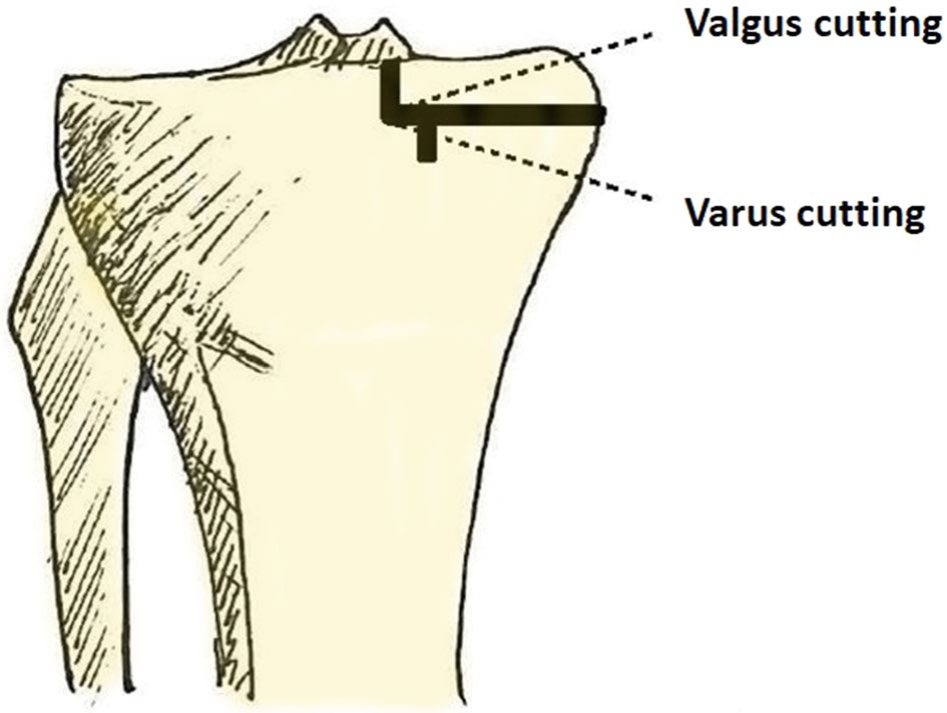
Illustration depicting the orientation of the tibial plateau cut. Varus cutting of the tibial plateau means more bone resection, whereas valgus cutting of the tibial plateau means less bone resection.

The tibial component alignment angle changes with the orientation of the tibial plateau resection during surgery. Varus cutting of the tibial plateau means more bone resection, which can lead to postoperative varus alignment in the lower limbs when a meniscal bearing with insufficient thickness is used to restore the joint space of the medial compartment. By contrast, valgus cutting of the tibial plateau may result in postoperative valgus alignment in the lower limb (Fig. 2). According to the results, the tibial component alignment angle significantly affected the postoperative HKA angle. The surgeon can intentionally adjust the orientation of the tibial plateau resection during surgery to affect tibial component alignment angle and postoperative lower limb alignment. However, when altering the coronal alignment of the tibial component, the biomechanics and stress distribution in the proximal tibia and the knee also change^42–45^. Sekiguchi et al. employed a musculoskeletal computer simulation program and discovered that different tibial component alignments displayed significantly different knee kinematics^42^. A three-dimensional finite element analysis showed that tibial component alignment affected the stress distribution in the proximal tibia^43–45^. Although many studies have provided recommendations for tibial component alignment, a consensus on the optimal angle has not been reached^42,46–49^. The majority of these studies suggest a slight varus placement of the tibial component, as it increases the keel-cortex distance, reduces medial/lateral translation, and enhances maximum total point motion, consequently reducing the potential risk of fracture, instability, and component loosening^42,46–48^. However, some studies suggest a slight valgus placement of the tibial component to reduce cancellous bone stresses^49^. To prevent poor postoperative outcomes, surgeons should pay attention to excessive varus or valgus malposition of the tibial component when adjusting its position^50,51^.

This study is the first to demonstrate that BMI had a significant effect on the postoperative HKA angle in the Oxford MUKA, indicating that more obese patients are prone to postoperative lower limb valgus alignment. Similarly, previous studies have indicated the effect of BMI on lower limb alignment and the femorotibial angles were different between obese and nonobese patients after TKA. Overweight has also been reported to cause postoperative lower limb malalignment^52–54^. Järvenpää et al. found that the obese group had significantly more cases of lower limb malalignment with a 3° deviation from the mechanical axis after TKA than the nonobese group^55^. Possible reasons include the impact of excessive fat on the accurate placement of the extramedullary alignment guide and cutting jig, potentially hindering exposure and bony landmarks. On the other hand, obesity can exert a multifactorial influence on knee osteoarthritis. In addition to causing excessive and abnormal joint loading, leading to wear on articular cartilage and bone, it can also affect surrounding tissues through the action of inflammatory cytokines^56^. This impact persists both preoperatively and postoperatively, continuously affecting lower limb alignment. Previous literature has also mentioned that obesity indeed contributes to a higher prevalence of lower limb valgus alignment in certain populations^57^. However, this study did not further analyze the differences in lower limb alignment between obese and non-obese individuals, which remains a topic for future investigation.

Robotic technology has emerged as a valuable tool for enhancing precision and outcomes in UKA. The primary advantage lies in facilitating meticulous preoperative planning and execution, overcoming complex anatomical variations encountered during surgery. Certain robotic systems provide real-time feedback, enabling intraoperative adjustments to optimize the positioning of components. This contributes to achieving more favorable postoperative lower limb alignment and component alignment in UKA^58^. While ongoing debate surrounds the long-term impact of robotic technology on implant survival, there is a consensus that robotic assistance reduces postoperative pain and accelerates recovery times^58–60^. However, most surgeons still need to be proficient in manual techniques and should be aware of factors that may impact postoperative lower limb alignment, especially in scenarios where robotic technology may not be readily available.

This study has some limitations. Firstly, the study was conducted at a single institution, which could limit the diversity of the patient population, introducing the potential for selection bias and thereby limiting generalizability. Secondly, some factors (e.g., the thickness of tibial plateau resections) were not recorded due to the retrospective nature of this study. This lack of information introduces the possibility of confounding bias. The amount of bone loss in the medial compartment may also affect the postoperative HKA angle. A prospective study should be conducted to analyze the effect of the thickness of the tibial plateau resection on postoperative lower limb alignment. Thirdly, full-length standing anteroposterior radiographs can be affected by the rotation of the lower limbs and the flexion contracture of the knees. Increased internal rotation of the knee joints results in a more valgus appearance of the leg axis, while external rotation leads to a corresponding increase in varus^61^. This phenomenon is particularly pronounced when flexion contracture occurs simultaneously^62^. Although efforts were made to exclude patients with poor-quality radiographs or difficult-to-measure deformities, the complete elimination of the effect of this factor could not be achieved. Fourthly, this study did not consider the factor of time, as lower limb alignment may change over an extended period due to the progression of the disease. However, this study opted to measure radiographs closest to the surgical date to minimize the influence of time on lower limb alignment. Finally, the effect of the postoperative HKA angle on clinical outcomes was not analyzed in this study. However, evaluation of the clinical outcomes was not the main purpose of this study. Future studies are advised to explore this relationship.

## Conclusions

In conclusion, postoperative HKA angle was affected by the following factors in descending order: preoperative HKA angle, bearing size, tibial component alignment angle, and BMI. Larger preoperative varus deformity, smaller bearing size, greater varus alignment of tibial component, and smaller BMI led to greater postoperative varus alignment of the lower limb. Keeping these concepts in mind, surgeons can more accurately predict postoperative lower limb alignment before and during surgery and thus attempt to minimize lower limb malalignment after Oxford MUKA. Future research can incorporate the temporal factor into analysis, exploring the continuous changes in lower limb alignment over time, and assessing their impact on clinical outcomes.

## Data Availability

The authors are able to provide the data that support the findings of this study upon request. Please contact the corresponding author for further information.

## References

[1] Matharu GS, Culliford DJ, Blom AW, Judge A (2022). Projections for primary hip and knee replacement surgery up to the year 2060: An analysis based on data from The National Joint Registry for England, Wales, Northern Ireland and the Isle of Man. Ann. R. Coll. Surg. Engl..

[2] Langdown AJ (2005). Oxford medial unicompartmental arthroplasty for focal spontaneous osteonecrosis of the knee. Acta. Orthop..

[3] Pandit H (2015). The clinical outcome of minimally invasive Phase 3 Oxford unicompartmental knee arthroplasty: A 15-year follow-up of 1000 UKAs. Bone Joint J..

[4] Walton NP (2006). Patient-perceived outcomes and return to sport and work: TKA versus mini-incision unicompartmental knee arthroplasty. J. Knee Surg..

[5] Lim JW, Cousins GR, Clift BA, Ridley D, Johnston LR (2014). Oxford unicompartmental knee arthroplasty versus age and gender matched total knee arthroplasty - functional outcome and survivorship analysis. J. Arthroplasty..

[6] Mohammad HR, Strickland L, Hamilton TW, Murray DW (2018). Long-term outcomes of over 8,000 medial Oxford Phase 3 Unicompartmental Knees-a systematic review. Acta. Orthop..

[7] Cao Z (2019). Comparison of Fixed-Bearing and Mobile-Bearing Unicompartmental Knee Arthroplasty: A Systematic Review and Meta-Analysis. J. Arthroplasty..

[8] Gulati A (2009). The effect of leg alignment on the outcome of unicompartmental knee replacement. J. Bone Joint Surg. Br..

[9] Petterson SC, Blood TD, Plancher KD (2020). Role of alignment in successful clinical outcomes following medial unicompartmental knee arthroplasty: Current concepts. J. ISAKOS..

[10] Hirschmann MT (2019). Functional knee phenotypes: A novel classification for phenotyping the coronal lower limb alignment based on the native alignment in young non-osteoarthritic patients. Knee Surg. Sports Traumatol. Arthrosc..

[11] Mullaji AB, Shetty GM, Kanna R (2011). Postoperative limb alignment and its determinants after minimally invasive Oxford medial unicompartmental knee arthroplasty. J. Arthroplasty..

[12] Mullaji AB, Shah S, Shetty GM (2017). Mobile-bearing medial unicompartmental knee arthroplasty restores limb alignment comparable to that of the unaffected contralateral limb. Acta. Orthop..

[13] Zhang Q (2018). FTFA change under valgus stress force radiography is useful for evaluating the correctability of intra-articular varus deformity in UKA. Arch. Orthop. Trauma Surg..

[14] Zhang Q (2019). Risk factors of postoperative valgus malalignment in mobile-bearing medial unicompartmental knee arthroplasty. Arch. Orthop. Trauma Surg..

[15] Ridgeway SR, McAuley JP, Ammeen DJ, Engh GA (2002). The effect of alignment of the knee on the outcome of unicompartmental knee replacement. J. Bone Joint Surg. Br..

[16] Hernigou P, Deschamps G (2004). Alignment influences wear in the knee after medial unicompartmental arthroplasty. Clin. Orthop. Relat. Res..

[17] Collier MB, Eickmann TH, Sukezaki F, McAuley JP, Engh GA (2006). Patient, implant, and alignment factors associated with revision of medial compartment unicondylar arthroplasty. J. Arthroplasty..

[18] Bruni D (2010). Minimally invasive unicompartmental knee replacement: Retrospective clinical and radiographic evaluation of 83 patients. Knee Surg. Sports Traumatol. Arthrosc..

[19] Slaven SE (2020). The impact of coronal alignment on revision in medial fixed-bearing unicompartmental knee arthroplasty. J. Arthroplasty..

[20] Kim SJ, Bae JH, Lim HC (2012). Factors affecting the postoperative limb alignment and clinical outcome after Oxford unicompartmental knee arthroplasty. J. Arthroplasty..

[21] Ji SJ (2021). Multivariate analysis of varus after Oxford unicompartmental knee arthroplasty. Beijing Da Xue Xue Bao Yi Xue Ban..

[22] Ramazanian T (2022). Distribution and correlates of hip-knee-ankle angle in early osteoarthritis and preoperative total knee arthroplasty patients. J. Arthroplast..

[23] Goodfellow JW, Kershaw CJ, Benson MK, O’Connor JJ (1988). The Oxford knee for unicompartmental osteoarthritis. The first 103 cases. J. Bone Joint Surg. Br..

[24] Svard UC, Price AJ (2001). Oxford medial unicompartmental knee arthroplasty. A survival analysis of an independent series. J. Bone Joint Surg. Br..

[25] Bitsch RG, Keudell, A.v, Losina, E. & Fitz, W. (2013). Good accuracy of the phase III Oxford mobile bearing unicompartmental knee instrumentation. Acta. Orthop..

[26] Marx RG (2011). Reliability of lower extremity alignment measurement using radiographs and PACS. Knee Surg. Sports Traumatol. Arthrosc..

[27] Moreland JR, Bassett LW, Hanker GJ (1987). Radiographic analysis of the axial alignment of the lower extremity. J. Bone Joint Surg. Am..

[28] Kennedy WR, White RP (1987). Unicompartmental arthroplasty of the knee. Postoperative alignment and its influence on overall results. Clin. Orthop. Relat. Res..

[29] Kerens B, Schotanus MG, Boonen B, Kort NP (2015). No radiographic difference between patient-specific guiding and conventional Oxford UKA surgery. Knee Surg. Sports Traumatol. Arthrosc..

[30] Park KK (2019). Robot-assisted unicompartmental knee arthroplasty can reduce radiologic outliers compared to conventional techniques. PLoS One..

[31] Manzotti A, Cerveri P, Pullen C, Confalonieri N (2014). Computer-assisted unicompartmental knee arthroplasty using dedicated software versus a conventional technique. Int. Orthop..

[32] Cohen, J. *Statistical Power Analysis for the Behavioral Sciences*(2nd ed.): Routledge New York (1988).

[33] Wen PF (2017). Effects of lower limb alignment and tibial component inclination on the biomechanics of lateral compartment in unicompartmental knee arthroplasty. Chin. Med. J. (Engl)..

[34] Sarangi PP, Karachalios T, Jackson M, Newman JH (1994). Patterns of failed internal unicompartmental knee prostheses, allowing persistence of undercorrection. Rev. Chir. Orthop. Reparatrice Appar. Mot..

[35] Vasso M (2015). Minor varus alignment provides better results than neutral alignment in medial UKA. Knee..

[36] Zuiderbaan HA (2016). Predictors of subjective outcome after medial unicompartmental knee arthroplasty. J. Arthroplasty..

[37] Tashiro Y (2014). The coronal alignment after medial unicompartmental knee arthroplasty can be predicted: Usefulness of full-length valgus stress radiography for evaluating correctability. Knee Surg. Sports Traumatol. Arthrosc..

[38] Hopgood P, Martin CP, Rae PJ (2004). The effect of tibial implant size on post-operative alignment following medial unicompartmental knee replacement. Knee..

[39] Lim HC, Bae JH, Song SH, Kim SJ (2012). Oxford phase 3 unicompartmental knee replacement in korean patients. J. Bone Joint Surg. Br..

[40] Kim SJ, Postigo R, Koo S, Kim JH (2014). Causes of revision following Oxford phase 3 unicompartmental knee arthroplasty. Knee Surg. Sports Traumatol. Arthrosc..

[41] Ahn JH, Kang HW, Yang TY, Lee JY (2016). Risk factors of post-operative malalignment in fixed-bearing medial unicompartmental knee arthroplasty. Int. Orthop..

[42] Sekiguchi K (2019). Effect of tibial component alignment on knee kinematics and ligament tension in medial unicompartmental knee arthroplasty. Bone Joint Res..

[43] Sawatari T, Tsumura H, Iesaka K, Furushiro Y, Torisu T (2005). Three-dimensional finite element analysis of unicompartmental knee arthroplasty-the influence of tibial component inclination. J Orthop. Res..

[44] Innocenti B, Pianigiani S, Ramundo G, Thienpont E (2016). Biomechanical effects of different varus and valgus alignments in medial unicompartmental knee arthroplasty. J. Arthroplast..

[45] Dai X (2018). How does the inclination of the tibial component matter? A three-dimensional finite element analysis of medial mobile-bearing unicompartmental arthroplasty. Knee..

[46] Barbadoro P (2014). Tibial component alignment and risk of loosening in unicompartmental knee arthroplasty: A radiographic and radiostereometric study. Knee Surg. Sports Traumatol. Arthrosc..

[47] Suda Y (2022). Varus placement of the tibial component of Oxford unicompartmental knee arthroplasty decreases the risk of postoperative tibial fracture. Bone Joint J..

[48] Kamenaga T (2024). Varus placement of the tibial component reduces the potential risk of fracture with adequate bony coverage in the Oxford unicompartmental knee arthroplasty. Sci. Rep..

[49] Iesaka K (2002). The effects of tibial component inclination on bone stress after unicompartmental knee arthroplasty. J. Biomech..

[50] Chatellard R (2013). Medial unicompartmental knee arthroplasty: Does tibial component position influence clinical outcomes and arthroplasty survival?. Orthop. Traumatol. Surg. Res..

[51] Kamenaga T, Hiranaka T, Hida Y, Fujishiro T, Okamoto K (2018). Effect of tibial component position on short-term clinical outcome in Oxford mobile bearing unicompartmental knee arthroplasty. J. Orthop. Sci..

[52] Estes CS, Schmidt KJ, McLemore R, Spangehl MJ, Clarke HD (2013). Effect of body mass index on limb alignment after total knee arthroplasty. J. Arthroplasty..

[53] Krushell RJ, Fingeroth RJ (2007). Primary total knee arthroplasty in morbidly obese patients: A 5- to 14-year follow-up study. J. Arthroplasty..

[54] Yoo JH, Oh HC, Park SH, Kim JK, Kim SH (2018). Does obesity affect clinical and radiological outcomes in minimally invasive total knee arthroplasty? Minimum 5-year follow-up of minimally invasive TKA in obese patients. Clin. Orthop. Surg..

[55] Jarvenpaa J, Kettunen J, Kroger H, Miettinen H (2010). Obesity may impair the early outcome of total knee arthroplasty. Scand. J. Surg..

[56] Chen L (2020). Pathogenesis and clinical management of obesity-related knee osteoarthritis: Impact of mechanical loading. J. Orthop. Translat..

[57] Bout-Tabaku S (2015). Obesity is associated with greater valgus knee alignment in pubertal children, and higher body mass index is associated with greater variability in knee alignment in girls. J. Rheumatol..

[58] Begum FA (2020). Robotic technology: Current concepts, operative techniques and emerging uses in unicompartmental knee arthroplasty. EFORT Open Rev..

[59] Crizer MP (2021). Robotic assistance in unicompartmental knee arthroplasty results in superior early functional recovery and is more likely to meet patient expectations. Adv. Orthop..

[60] Gaudiani MA (2022). 5-Year Survivorship and Outcomes of Robotic-Arm-Assisted Medial Unicompartmental Knee Arthroplasty. Appl. Bionics Biomech..

[61] Jamali AA (2017). Do small changes in rotation affect measurements of lower extremity limb alignment?. J. Orthop. Surg. Res..

[62] Brunner J (2023). Significant changes in lower limb alignment due to flexion and rotation-a systematic 3D simulation of radiographic measurements. Knee Surg. Sports Traumatol. Arthrosc..

